# Colonic Mucosal Microbiota and Association of Bacterial Taxa with the Expression of Host Antimicrobial Peptides in Pediatric Ulcerative Colitis

**DOI:** 10.3390/ijms21176044

**Published:** 2020-08-22

**Authors:** Jonna Jalanka, Jing Cheng, Kaisa Hiippala, Jarmo Ritari, Jarkko Salojärvi, Tarja Ruuska, Marko Kalliomäki, Reetta Satokari

**Affiliations:** 1 Human Microbiome Research Program, Faculty of Medicine, University of Helsinki, P.O. Box 21, FI-00014 Helsinki, Finland; jonna.jalanka@helsinki.fi (J.J.); hallen003@hotmail.com (J.C.); Kaisa.Hiippala@helsinki.fi (K.H.); 2 Finnish Red Cross Blood Service, 00310 Helsinki, Finland; Jarmo.Ritari@veripalvelu.fi; 3 School of Biological Sciences, Nanyang Technological University, 60 Nanyang Drive, Singapore 637551, Singapore; jarkko.salojarvi@helsinki.fi; 4 Department of Pediatrics, University of Tampere and Tampere University Hospital, P.O. Box 2000, 33521 Tampere, Finland; tarja.ruuska@sci.fi; 5 Department of Pediatrics, University of Turku and Turku University Central Hospital, P.O. Box 52, 20521 Turku, Finland; markal@utu.fi; 6 Functional Foods Forum, University of Turku, 20014 Turku, Finland

**Keywords:** inflammatory bowel disease, ulcerative colitis, microbiota, gene expression, host-microbe cross-talk

## Abstract

Inflammatory bowel diseases (IBD), ulcerative colitis (UC) and Crohn’s disease (CD), are chronic debilitating disorders of unknown etiology. Over 200 genetic risk loci are associated with IBD, highlighting a key role for immunological and epithelial barrier functions. Environmental factors account for the growing incidence of IBD, and microbiota are considered as an important contributor. Microbiota dysbiosis can lead to a loss of tolerogenic immune effects and initiate or exacerbate inflammation. We aimed to study colonic mucosal microbiota and the expression of selected host genes in pediatric UC. We used high-throughput 16S rDNA sequencing to profile microbiota in colonic biopsies of pediatric UC patients (*n* = 26) and non-IBD controls (*n* = 27). The expression of 13 genes, including five for antimicrobial peptides, in parallel biopsies was assessed with qRT-PCR. The composition of microbiota between UC and non-IBD differed significantly (PCoA, *p* = 0.001). UC children had a decrease in Bacteroidetes and an increase in several family-level taxa including Peptostreptococcaceae and Enterobacteriaceae, which correlated negatively with the expression of antimicrobial peptides REG3G and DEFB1, respectively. Enterobacteriaceae correlated positively with the expression siderophore binding protein LCN2 and Betaproteobacteria negatively with DEFB4A expression. The results indicate that reciprocal interaction of epithelial microbiota and defense mechanisms play a role in UC.

## 1. Introduction

Inflammatory bowel diseases (IBD) affect up to seven million people globally and three million in Europe and their incidence and prevalence are increasing [1,2]. The two main conditions of IBD are ulcerative colitis (UC) and Crohn’s disease (CD). Epidemiological patterns suggest that IBD will emerge as a major worldwide disease in the coming years [1,2]. The etiology of IBD is multifactorial and the course of pathogenesis is still largely unknown. Genome-wide association studies (GWAS) have identified over 200 genetic loci as genetic risk factors for IBD [3] and more recently, epigenetic alternations were described in the epithelium of IBD subjects [4]. The majority of the IBD-associated genes code for immunological and epithelial barrier functions, which direct the host´s response to environmental factors and consequently determine mucosal homeostasis [3]. Furthermore, both omics-based and targeted analysis of gene expression in the intestinal epithelium have revealed IBD-associated alterations, which were independent from inflammation status [4,5]. Antimicrobial peptides (AMPs), including α- and β-defensins, C type lectins, cathelicidins and S100 proteins, are widely produced as a defense mechanism by intestinal epithelial cells to regulate and maintain homeostasis between the microbiota and the host epithelium [6]. The expression of AMPs by small intestinal Paneth cells, mainly α-defensins, is altered in ileal CD resulting in reduced antimicrobial activity, whereas in UC the colonic antimicrobial barrier, formed by a mucus layer retaining the AMPs, is impaired despite the upregulated epithelial peptide production [6,7].

The growing incidence of IBD suggests that environmental factors that have changed during the past decades, such as urbanization, hygienic conditions and microbial exposure, use of chemicals, diet, smoking and the use of antibiotics and other medication, modify the disease risk of genetically predisposed individuals [1,2,8]. Microbiota are a major dictator of the antigenic milieu in the intestine and also have a regulatory effect on the host gene expression in the mucosa, and therefore can affect immunological balance in the gut epithelium [9,10,11,12]. Both fecal and mucosal microbiota profiles of IBD patients have been found to differ from those of healthy subjects, and microbiota with abnormal composition, i.e., dysbiotic microbiota, are characteristic to the disease. The key findings include reduced bacterial diversity, richness and stability [11,13,14,15], depletion of immune-regulatory species, such as *Akkermansia muciniphila* [14] and *Faecalibacterium prausnitzii* and other butyrate-producing bacteria [14,16], and increase in proinflammatory Proteobacteria, especially Enterobacteriaceae [10,17]. Overall, the depletion of core gut commensals, Bacteroidales and Clostridiales, is characteristic to IBD and the disease-associated changes are more prominent in mucosa than feces [17,18]. Although it has not been clarified whether microbiota perturbation is the cause or the result of inflammation, it seems justified to consider the microbiota as one of the key players in IBD etiopathology. Furthermore, several recent studies have indicated that microbiota profiles of treatment-naïve IBD patients are predictive for the disease course and treatment responsiveness [18,19].

In Finland, the prevalence of IBD is high and the incidence of pediatric IBD increased up to 8% per year in the early 2000s [20,21]. Registry-based cohort studies from both Finland and Sweden showed that repeated use of antibiotics, especially cephalosporins, in childhood has been associated with an increased risk of developing IBD [20,22], which may be due to the changes in the microbiota. Kolho and colleagues studied the fecal microbiota in Finnish pediatric IBD patients and found reduced bacterial richness and abundance of butyrate producers [21]. As mucosa-associated microbiota dysbiosis could be more obvious and relevant for inflammation, we focused in this study on the mucosa-associated microbiota of Finnish pediatric patients with ulcerative colitis (UC, *n* = 26) and non-IBD subjects as (healthy) controls (non-IBD, *n* = 27). In addition, we studied the mucosal expression of selected genes related to epithelial barrier function and coding for antimicrobial peptides or receptors for bacterial recognition.

## 2. Results

### 2.1. Study Cohort and Effect of Sampling on the Microbiota

Clinical characteristics of the study groups are presented in Table 1. According to the Mayo endoscopic subscore, 8 of the UC patients had inactive and 18 had active disease. The biopsies were collected from different parts of the large intestine, i.e., cecum, ascending and descending colon, and therefore we first investigated the possible effect of biopsy location on the microbiota composition. The PCoA of all samples, both UC and non-IBD, indicated that biopsy location has a significant impact on the microbiota composition, although the subgroup analysis of UC and non-IBD did not separately show the effect of location (Supplementary Figure S1). Further, the patient age had a significant effect on the microbiota composition (Supplementary Figure S2). Based on these results, the sampling location and age were included as confounders in all subsequent analysis.

### 2.2. Mucosal Microbiota Associated with UC

In assessing the UC-associated microbiota, we first compared microbiota diversity and richness between active, inactive disease and non-IBD, and found no significant difference between the groups (Supplementary Figure S3).

**Figure 1:**
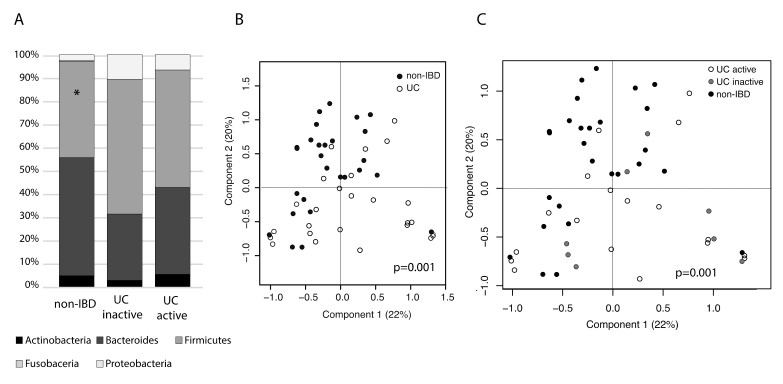
Colonic mucosal microbiota composition in ulcerative colitis (UC) subjects with active and inactive disease compared to non-inflammatory bowel diseases (IBD) controls. (**A**) Bacterial phylum level composition. Non-IBD controls had significantly less Firmicutes than UC patients (*p* = 0.006). (**B**) PCoA on bacterial family level taxa and sample annotations to UC vs. non-IBD. (**C**) PCoA on bacterial family-level taxa and sample annotations to UC active, UC inactive and non-IBD.

Next, we compared the microbiota composition at bacterial phylum level. Non-IBD subjects were found to harbor significantly less Firmicutes (Figure 1A, *p* = 0.006) than UC patients, as well as more Bacteroidetes and less Proteobacteria as compared to UC, although the latter differences were not statistically significant. Subsequent analysis with higher taxonomic resolution separated UC and non-IBD in PCoA at bacterial family level (Figure 1B, *p* = 0.001) and disease activity accounted for 9% of variation in the data (Figure 1C, *p* = 0.001).

**Figure 2:**
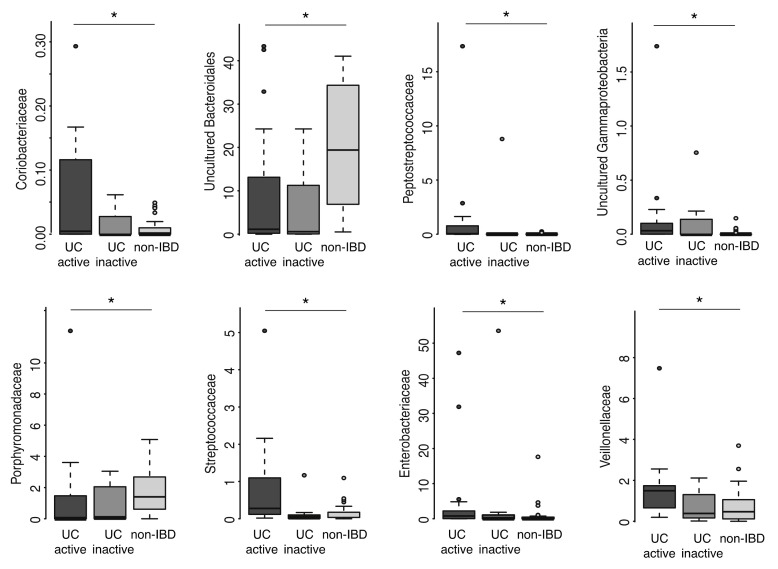
Family-level microbiota differences between active, inactive ulcerative colitis and non-IBD controls. Microbiota abundance expressed as relative abundance. An asterisk indicates statistical significance between active UC and non-IBD control.

Concerning individual bacterial taxa at family level, Sutterellaceae, Veillonellaceae and unclassified Erysipelotrichia were increased, and unclassified Bacteroidetes and Negativicutes decreased in UC (Table 2). Further subgroup analysis revealed interesting patterns concerning increasing and decreasing abundances of individual taxa from active to inactive UC and to non-IBD (Figure 2). Most notably, unclassified Bacteroidales and Porhyromonadaceae (also belonging to the Bacteroidales order) as well as unclassified Bacteroidia were decreased in UC as compared to non-IBD, whereas Coriobacteriaceae, Streptococcaceae, Peptostreptococcaceae, Veillonellaceae, Enterobacteriaceae and unclassified Gammaproteobacteria were increased in UC. Inactive UC differed from active disease in having a decreased abundance of Streptococcaceae and Peptostreptococcaceae, and an increased abundance of unclassified Saccharibacteria (formerly known as TM7 phylum), which was equally low in abundance in active UC and non-IBD. Across all samples, Saccharibacteria correlated positively with Micrococcaceae (r = 0.35, *p* = 0.01) and Ruminococcaceae (r = 0.28, *p* = 0.04).

### 2.3. Mucosal Gene exPression and Correlations with Microbial Taxa

The relative gene expression of 6 out of the 13 studied genes differed between UC subjects and controls, and also between active and inactive disease (Table 3). The expression of interleukin 8 (IL-8), chemokine CXCL16, the calcium binding proteins S100A8 and S100A9 and lipocalin 2 (LCN2) were significantly increased and the expression of antimicrobial peptide DEFB1 was significantly decreased in UC patients when compared to non-IBD. The expression of the other studied genes for antimicrobial peptides DEFB103B, DEFB4A, RETNLB and REG3G did not differ between the study groups, although the last mentioned showed a tendency for decreased expression in UC (*p* = 0.076). Further, the expression of trefoil factor 3 (TFF3), which is involved in the maintenance and repair of intestinal mucosa, and the main colonic mucin MUC2 were at similar levels in both study groups. Correlation analysis of the microbial and qRT-PCR data revealed correlations between the abundance of family-level taxa and the expression of specific genes (Table 3). The abundance of Peptostreptococcaceae and Enterobacteriaceae correlated negatively with the expression of antimicrobial peptides REG3g and DEFB1, respectively. The abundance of Enterobacteriaceae correlated positively with the expression of siderophore binding protein LCN2 and Betaproteobacteria correlated negatively with the expression of DEFB4A. Sutterellaceae and Veillonellaceae had positive correlations with the expression of CXCL16 chemokine and its receptor CXCR6 as well as IL-8. The abundance of Lactobacillaceae correlated negatively with the expression of CXCL16, S100A8 and S100A9.

## 3. Discussion

Previous studies have suggested that both microbiota and impaired function of the intestinal epithelium contribute to UC pathogenesis. Here, we carried out high-throughput sequencing of the microbiota and applied a targeted host gene expression analysis to study mucosal microbiota–host interactions in Finnish pediatric UC patients and controls. The main limitation of our study is that the cohort size is relatively small and hence some of the results, particularly those showing associations between microbial abundance with mucosal gene expressions, should be considered as preliminary, and confirmed in a larger group of study subjects. Moreover, there was variation in the subjects’ age and biopsy location, which was taken into account in the microbiota analyses. Unfortunately, we did not gather long-term information on the history of antibiotic usage before the sampling, and it was not possible to assess the impact of overall antibiotic use on microbiota in this cohort, although at the time of sampling the study subjects were not receiving antibiotics.

On the other hand, the homogenous ethnic background of the study population could be considered as a strength. Our cohort included children with varying ages and biopsy locations, and both of these factors were included as confounders in the microbiota analysis. Similar to our results, age has been found to have a significant impact on the composition of both mucosal and fecal microbiota in children [18,23]. Concerning biopsy location, our results were not fully conclusive that mucosal microbiota would significantly differ between different parts of the colon, but for prudence it was included as a confounder. Some previous studies have concluded that mucosal microbiota differs significantly between colon segments [4], whereas others have considered it to be fairly comparable [17].

Our results on the comparison of microbiota between patients having active and inactive UC and control subjects reassert the previously described UC-associated dysbiosis that has been characterized by the depletion of anaerobic commensals and increase in facultatively anaerobic taxa [10,18], while bacterial diversity or richness may not be affected [4]. We found depletion of Bacteroidetes and several family-level taxa in the colonic mucosa of pediatric UC patients, which has also been described for pediatric CD [17]. *Bacteroides* species, albeit also being opportunistic pathogens, are considered as health-promoting in the gut mucosa, as they are capable of reinforcing the epithelial barrier, exerting anti-inflammatory actions by releasing polysaccharide A (PSA), sphingolipids and outer membrane vesicles, and ameliorating experimental colitis [24,25,26].

We observed that UC patients had an increased amount of Enterobacteriaceae and other unclassified Gammaproteobacteria, Sutterellaceae, Veillonellaceae, Streptococcaceae and Peptostreptococcaceae, which was emphasized in the patients with active disease. This replicates previous studies showing these taxa to be increased in pediatric CD or UC [17,18], and particularly Gammaproteobacteria and Enterobacteriaceae are renowned for their proinflammatory properties due to the production of lipopolysaccharide (LPS) [9,10]. Veillonellaceae and specific species within Streptococcaceae have also been previously linked with more severe disease progression in new-onset pediatric UC [18]. Concerning Sutterellaceae, the association with IBD is unclear as the results vary between studies [17,18,27]. All these families, excluding Peptostreptococcaceae, are aerotolerant and increased oxygen levels in the inflamed gut may promote their growth, as suggested by the oxygen hypothesis [28]. Peptostreptococcaceae are anaerobic commensals, whose increased abundance in UC may be linked to other factors than increased oxygen levels during inflammation, such as altered expression of antimicrobial peptides in the epithelium, which is supported by the result that Peptostreptococcaceae abundance correlated negatively with the expression of *REG3G*. The expansion of this bacterial taxa has been previously observed in adult UC patients in remission [14].

A novel finding in our study was that patients with inactive UC had an increased abundance of Saccharibacteria as compared to non-IBD and active UC patients, and that across all samples Saccharibacteria correlated positively with Micrococcaceae and Ruminococcaceae, the latter of which has been found to be reduced in treatment-naïve pediatric UC and CD [17,18]. Saccharibacteria (formerly known as the TM7 phylum) are ultrasmall bacteria, which parasitize on other bacteria and display highly dynamic interactions with their hosts, including virulent killing [29]. Thus, Saccharibacteria may affect the gut microbiota structure and functionality, and consequently mucosal homeostasis. For example, specific species of Saccharibacteria have been shown to silence the ability of its host bacterium to induce TNF-alpha expression in macrophages [30]. Thereby, our finding on the increased abundance of Saccharibacteria in inactive UC is of particular interest, and the possible involvement of this bacterial group in fluctuating microbiota composition and remission–relapse cycling in UC should be studied further.

Overall, it seems that the shifts in the balance of the mucosal microbiota in UC towards increased proportions of pro-inflammatory bacteria, especially Enterobacteriaceae, and decreased proportions of anti-inflammatory bacteria, such as *Bacteroides* spp., may initiate and exacerbate inflammation. The origin of microbiota shifts is an intriguing question, and future studies should address microbiota changes in patients longitudinally across remission–relapse cycles and also investigate the possible role of less studied microbial groups in the shifts, including phages and the ultrasmall bacterial parasites Saccharibacteria that were found to be increased in UC in this study.

The quantitative expression analysis of 13 selected genes revealed differential expression of six genes in UC as compared to non-IBD. These included *IL-8* and *CXCL16*, which were previously found to have increased expression in pediatric UC patients [5], as well as the calcium binding proteins *S100A8* and *S100A9*, whose complex is also known as calprotectin—an established biomarker of disease activity in UC and other chronic inflammatory diseases. We also confirmed now in a pediatric cohort the previous findings of decreased expression of antimicrobial defensin β 1 (DEFB1), a key effector of the innate immune system [31]. Moreover, we showed that the decrease in expression could be linked to an increased Enterobacteriaceae abundance. Defective production of DEFB1 could potentially lead to the increased levels of proinflammatory bacteria and, therefore, activation of the mucosal immune system and activity of the inflammatory disease.

In addition to these findings, we showed that Sutterellaceae and Veillonellaceae had a positive correlation with the expression of *CXCL16* and although the causal link remains speculative, the possible regulatory functions of these taxa on mucosal *CXCL16* expression is an intriguing question that could be addressed in future studies. Interestingly, the expression of *CXCL16*, *S100A8* and *S100A9* correlated negatively with Lactobacillaceae, which are proposed to exert anti-inflammatory action in the gut [32]. However, we did not find correlation between the abundance of Lactobacillaceae and the expression of the selected AMP, although recent animal model studies have shown that *Lactobacillus* spp. could stimulate AMP production [33,34,35].

The negative correlation between the expression of antimicrobial peptides REG3g and DEFB1 and Peptostreptococcaceae and Enterobacteriaceae may partly explain the increase in abundance of these taxa in UC. On the other hand, Betaproteobacteria abundance correlated negatively with the expression of *DEFB4A*, and although *DEFB4A* expression did not differ between the study groups, the result supports the idea that a repertoire of antimicrobial peptides participates in maintaining microbiota eubiosis. However, the positive correlation between *LCN2* expression and Enterobacteriaceae may suggest that these bacteria induce the expression of the siderophore binding protein as a host defense mechanism to limit the availability of iron and subsequently bacterial growth.

In summary, our results reinforce the suggestion that reciprocal interaction of mucosal microbiota and epithelial defense mechanisms play a role in UC. The depletion of Bacteroidetes and increase in facultative anaerobes including Enterobacteriaceae may negatively affect the immunological tolerance towards gut microbiota. The negative correlation between the expression of antimicrobial peptides and specific taxa suggests that impaired host defense mechanisms may allow the expansion of specific microbes able to exacerbate inflammation in UC. The results provide leads for further studies to investigate host–microbiota interactions and can help to develop strategies to restore mucosal microbiota and homeostasis in UC.

## 4. Materials and Methods

### 4.1. Patients

Patients were recruited at the Department of Pediatrics in Turku University Hospital, Turku, Finland and Tampere University Hospital, Tampere, Finland. Endoscopies were done on the clinical basis due to a previous diagnosis of UC or symptoms suggestive of IBD. Endoscopic findings in colonoscopy were classified according to the Mayo endoscopic subscore as normal or inactive disease (score 0), mild (score 1), moderate (score 2) and severe disease (score 3) [36]. The division of the patients to the active and inactive UC groups was based on the histological scoring, which was in accordance with the endoscopic Mayo scoring. The following groups of patients were included in the study: children with endoscopically active UC (*n* = 18), children with clinically and endoscopically quiescent UC (*n* = 8) and 27 children with macroscopically and microscopically non-inflamed colon to whom endoscopy was done due to various reasons (Table 1), such as chronic diarrhea (*n* = 7), abdominal pain (*n* = 8), hematochezia (*n* = 9) or other gastrointestinal symptoms (*n* = 3). These were included in the study as non-IBD controls. In children with active UC, biopsies were taken in the involved area, i.e., where macroscopic inflammation was found during endoscopy, and these locations included cecum and ascending and descending colon. In inactive UC and non-IBD subjects, biopsies were collected from the same locations to allow reasonable comparison between the groups. The study cohort demographics are presented in Table 1. The study subjects were not receiving antibiotics at the time of sampling or for four weeks prior to the sampling. Written informed consent was obtained from all the study patients or their parents. The study was accepted by the ethical committee of the hospital district of Southwest Finland.

### 4.2. Isolation of Host RNA and Microbial DNA

Two biopsies, one for microbial DNA extraction and one for RNA isolation, were taken from each patient, in addition to the routine biopsies for histological examination. The biopsy was taken in the involved area if macroscopic inflammation was found during endoscopy. Otherwise, the biopsy was taken in a non-involved area. In any case, a sample for histological evaluation was taken in the same area as the biopsy for RNA isolation in order to confirm whether the area was inflamed or not. The collected biopsies represented different locations, i.e., cecum, ascending and descending colon (Table 1). Biopsy samples for microbial DNA isolation were frozen in −80 °C within 2 h of collection, and the microbial DNA was extracted as described previously [37]. Biopsy samples for RNA analysis were rinsed with RNAse-free water and then immediately immersed in RNAlater RNA stabilization reagent (Qiagen, Hilden, Germany), then incubated at 4 °C for 1 day and stored at −20 °C until RNA isolation. After the tissue homogenization, isolation of RNA was performed by RNeasy Plus Mini-kit (Qiagen) according to the manufacturer’s instructions [37]. Quality of the isolated RNA was analyzed with Bio-Rad Experion System (Bio RAD Laboratories, Hercules, CA, USA).

### 4.3. 16.S rDNA Amplicon Sequencing

Amplicons from the V1 to V2 region of 16S rRNA genes were generated by PCR using the degenerated primers 27F-DegL (5′-AGRGTTYGATYMTGGCTCAG-3′) and 338R (5′-TGCTGCCTCCCGTAGGAGT-3′) producing a ~311 bp amplicon [38]. To facilitate pyrosequencing using titanium chemistry, each forward primer was appended with the titanium sequencing adaptor A and an “NNNNNNNN” barcode sequence at the 5′ end, where NNNNNNNN is a sequence of eight nucleotides that was unique for each sample. The reverse primer carried the titanium adaptor B at the 5′ end.

PCRs were performed using a Mx3005P thermocycler (Stratagene, La Jolla, CA, USA) in a total volume of 25 μL containing 1× PCR buffer, 1 μL PCR-grade nucleotide mix, 2.4 units of AmpliTaq Gold DNA polymerase (Applied Biosystems/Life Technologies Waltham, MA, USA), 200 nM forward and reverse primers (Oligomer, Helsinki, Finland) and 100 to 300 ng of template DNA. The amplification program consisted of an initial denaturation step at 96 °C for 2 min; 35 cycles of denaturation at 96 °C for 30 s, annealing at 56 °C for 45 s and elongation at 72 °C for 60 s; and a final extension step at 72 °C for 10 min. Three parallel reactions per sample were prepared. The size of the PCR products was confirmed by gel electrophoresis using 1% (wt/vol) agarose gel and ethidium bromide staining. Control PCRs were performed alongside each separate amplification without addition of template, and consistently yielded no product. PCR products from 3 to 5 parallel reactions were pooled and purified with the QIAquick PCR purification kit (Qiagen, Hilden, Germany) followed by DNA yield quantification using a NanoDrop ND-1000 spectrophotometer. The pooled amplicons were pyrosequenced using a 454-GS FLX titanium chemistry (Roche Diagnostics, Rotkreuz, Switzerland) in the sequencing core facility in the Institute of Biotechnology, University of Helsinki using the manufacturer´s protocols. Data published in ENA ref. no PRJEB38527.

### 4.4. qRT-PCR of Mucosal Gene Expression

Reverse transcription reactions were done by High Capacity cDNA reverse transcription kit (Applied Biosystems/Life Technologies Corporation, Carlsbad, CA, USA) as described previously [5,37]. Gene expression assays were performed according to the protocol using the comparative Ct (threshold cycle)-method with Applied Biosystems´ ABI 7300 Real Time PCR System and targeting the genes of interest based on previous literature. The following 14 genes were analyzed (Taqman Gene Expression Assay ID in brackets): *IL8* (Hs00174103_m1), *CXCL16* (Hs00222859_m1), *CXCR6* (Hs01890898_s1), *DEFB1* (Hs00608345_m1), *DEFB103B* (Hs04194486_g1), *DEFB4A* (Hs00823638_m1), *LCN2* (Hs01008571_m1), *MUC2* (Hs03005103_g1), *REGIIIg* (Hs01595406_g1), *RETNLB* (Hs00395669_m1), *S100A8* (Hs00374263_m1), *S100A9* (Hs00610058_m1), *TFF3* (Hs00902278_m1) and *18S* rRNA (Hs99999901_s1). The expression of the 18S rRNA gene was used as an endogenous control due to its constant expression in all the study samples [5]. Thermal cycler conditions were (1) 50 °C for 2 min, (2) 95 °C for 10 min, (3) 95 °C for 15 s and (4) 60 °C for 1 min, with 40 cycles of steps 3 and 4. Negative and positive (cDNA of the Universe Human Reference RNA, Agilent Technologies, Santa Clara, CA, USA) controls were included in all PCR runs. Results were analyzed with Applied Biosystems´ RQ-Study program and gene expressions relative to the positive control were calculated as previously described [5,37].

### 4.5. 16.S rDNA Amplicon Data Analysis

Pyrosequences were sorted per barcode. We processed 876,571 raw reads using in-house R scripts and the Quantitative Insights Into Microbial Ecology (QIIME) software package version 1.9 [39]. Preprocessing in R included removal of chimeric reads by mapping to the ChimeraSlayer reference database (Broad MIcrobiome Utilities version microbiomeutil-r20110519) using the Usearch v. 8.0.1623 uchime_ref algorithm with default settings [40,41]. Furthermore, sequences having length <300 nt were excluded. In QIIME, the preprocessing included removing reads lacking a barcode or primer sequence and removing the forward and reverse primer sequences from the reads. The quality control steps in QIIME were done with default settings. Briefly, a maximum of 6 ambiguous bases per read was allowed and sequences were discarded if the average quality score over a sliding window spanning 50 nucleotides dropped below 25 [39]. The final dataset included 393,237 reads, with a mean read count of 5869 per sample. The OTUs detected once across all samples were removed. OTUs for the filtered reads were defined at 97% sequence similarity using UCLUST in QIIME [40]. Representative sequences from each OTU were taxonomically assigned with the Uclust method and the SILVA v.119 reference database in QIIME.

### 4.6. Statistical Analysis

The data analysis was performed in R version 2.15.1 (R Development CT 2012) and by using in-house scripts. Microbial richness and the community diversity (Shannon diversity) and the proportion of how different parameters effect the variation (MANOVA) was calculated using functions from the Vegan package. Microbiota compositional analysis was conducted using MARE functions using the family taxonomical level [42]. Principal co-ordinate analysis (PCoA) was used to visualize the dissimilarities in the microbial community using Bray–Curtis dissimilarities.

The statistical difference in family-level taxa abundances between UC and non-IBD as well as UC active and inactive and non-IBD was tested with generalized linear mixed models with functions from the MARE R package. The read number for each sample was used as an offset to account for the varying sequencing depth and biopsy location, and sequencing patch and subjects’ age were used as confounding factors. The obtained *p*-values were corrected using multiple testing with the false discovery rate approach. The values with *p*-values below 0.01 and FDR-adjusted *p*-values (q-values) below 0.2 were considered to be significant. Associations between gene expression and bacterial abundances were estimated with generalized linear mixed models with functions from the MARE R package. The read number for each sample was used as an offset and the biopsy location and subjects’ age were used as confounding factors. The FDR-corrected *p*-values below 0.05 were considered to be significant.

The difference in phylum-level abundances and gene expression levels between groups were analyzed with ANOVA with Benjamini–Hochberg (BH) adjustment. The correlations between bacterial groups and gene expressions were estimated by Spearman coefficient, followed by FDR correction (BH) of *p*-value.

### 4.7. Ethical Considerations

The study was accepted by the ethical committee of the Hospital District of Southwest Finland (Journal number 16.11.2004 § 344). Written informed consent was obtained from all of the study patients or their parents.

## Figures and Tables

**Table 1:** Patient demographics.

	UC Active	UC Inactive	Control (HC)
Number of patients	*n* = 18	*n* = 8	*n* = 27
Age (range)	13 (5–17)	14 (10–16)	8 (3–15)
Biopsy collected			
ascending	8	3	2
cecum	4	4	17
descending	6	1	8
Mayo score			
0	-	8	27
1	11	-	-
2	5	-	-
3	2	-	-
Diagnosis or reason for diagnoctic colonoscopy			
Pancolitis	16	7	-
Left-sided colitis	2	1	-
Diabetes mellitus	-	-	2
Diarrhea	-	-	7
Asthma	-	-	1
Abdominal pain	-	-	8
Hematochezia	-	-	9
Medication			
None	7	0	24
5-ASA	11	8	-
Prednisolone	1	0	-
Azathioprine	4	3	-
Metotrexate	-	1	-
Insulin	-	-	2
Budesonide	-	-	1

**Table 2:** Family-level microbiota difference between ulcerative colitis (UC) patients and controls.

Taxon	Fold Change in UC as Compared to HC	*p*-Value	*q*-Value
Uncultured Bacteroidetes	0.58	0.003	0.06
Uncultured Erysipelotrichia	7.27	0.021	0.17
Uncultured Negativicutes	0.61	0.005	0.06
Veillonellaceae	2.93	0.013	0.13
Sutterellaceae	4.35	2.96E-07	1.15E-05

**Table 3:** Expression of selected genes and their associations with bacterial abundance.

	Fold Change in Gene Expression		Associations between Gene-Expression and Microbiota	
Gene Expression	UC/HC	UC Active/ UC Inactive	Negative	Positive
CXCL16	1.31 *	1.56 *	Lactobacillaceae	Veillonellaceae
CXCR6	ns.	ns.	-	Sutterellaceae, Veillonellaceae
DEFB1	0.76 *	0.34 ***	Enterobacteriaceae	-
DEFB103B	ns.	ns.	-	-
DEFB4A	ns.	ns.	Uncultured Betaproteobacteria	-
IL8	68.19 ***	38.36 ***	-	Sutterellaceae, Veillonellaceae
LCN2	6.32 **	7.59 **	-	Enterobacteriaceae
MUC2	ns.	ns.	-	Desulfovibrionaceae
REGIIIg	ns.	ns.	Peptostreptococcaceae	-
RETNLB	ns.	ns.	-	-
S100A8	18.78 **	29.30 *	Lactobacillaceae	Actinomycetaceae
S100A9	8.58 *	17.12 **	Lactobacillaceae	Actinomycetaceae
TFF3	ns.	ns.	-	-

* *p* < 0.05, ** *p* < 0.001, *** *p* < 0.0001.

## Supplementary Materials

Supplementary Materials can be found at https://www.mdpi.com/1422-0067/21/17/6044/s1.

## Abbreviations

IBD	Inflammatory bowel diseases
UC	Ulcerative colitis
CD	Crohn’s disease
16S rDNA	Small sub-unit ribosomal RNA gene
qRT-PCR	Quantitative reverse-transcriptase polymerase chain reaction
OTU	Operational taxonomic unit
PCoA	Principal co-ordinate analysis
FDR	False discovery rate
