# Supplementary material for: Colonic Mucosal Microbiota and Association of Bacterial Taxa with the Expression of Host Antimicrobial Peptides in Pediatric Ulcerative Colitis
Source: Int J Mol Sci. 2020 Aug 22;21(17):6044. doi: 10.3390/ijms21176044 (PMC7504357; doi:10.3390/ijms21176044)
Supplement: Supplementary file 1 [file ijms-21-06044-s001.zip › Supplementary figure 1.pdf]

Supplementary figure 1:

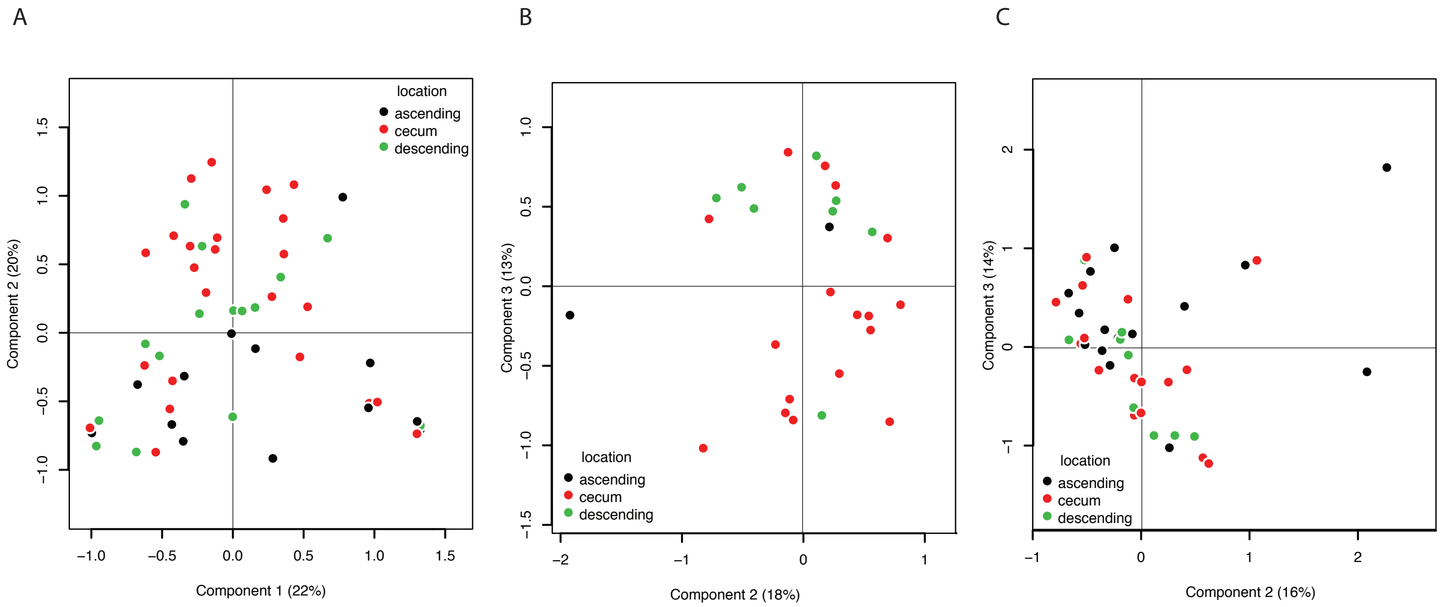

Supplementary Figure 1. The effect of location on microbial composition. **A)** When considering all patients the location has a significant effect (p=0.004) on the microbial composition, contributing 9% of the microbial variation. However, when assessing the study groups **B)** non-IBD (p=0.07) and **C)** UC patients (p=0.18) separately, the biopsy location did not have a significant effect on the microbial composition.
