# Supplementary material for: Colonic Mucosal Microbiota and Association of Bacterial Taxa with the Expression of Host Antimicrobial Peptides in Pediatric Ulcerative Colitis
Source: Int J Mol Sci. 2020 Aug 22;21(17):6044. doi: 10.3390/ijms21176044 (PMC7504357; doi:10.3390/ijms21176044)
Supplement: Supplementary file 1 [file ijms-21-06044-s001.zip › Supplementary figure 3.pdf]

Supplementary figure 3:

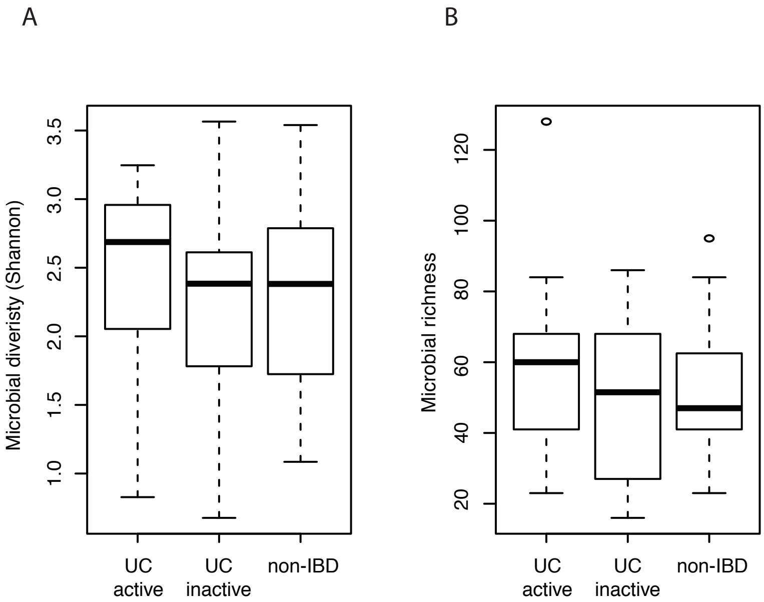

Supplementary Figure 3. Microbiota diversity (A) and richness (B) in the colonic mucosa of UC subjects with active and inactive disease compared to controls.
