# Supplementary material for: Colonic Mucosal Microbiota and Association of Bacterial Taxa with the Expression of Host Antimicrobial Peptides in Pediatric Ulcerative Colitis
Source: Int J Mol Sci. 2020 Aug 22;21(17):6044. doi: 10.3390/ijms21176044 (PMC7504357; doi:10.3390/ijms21176044)
Supplement: Supplementary file 1 [file ijms-21-06044-s001.zip › supplementary figure age.pdf]

Supplementary figure 2.

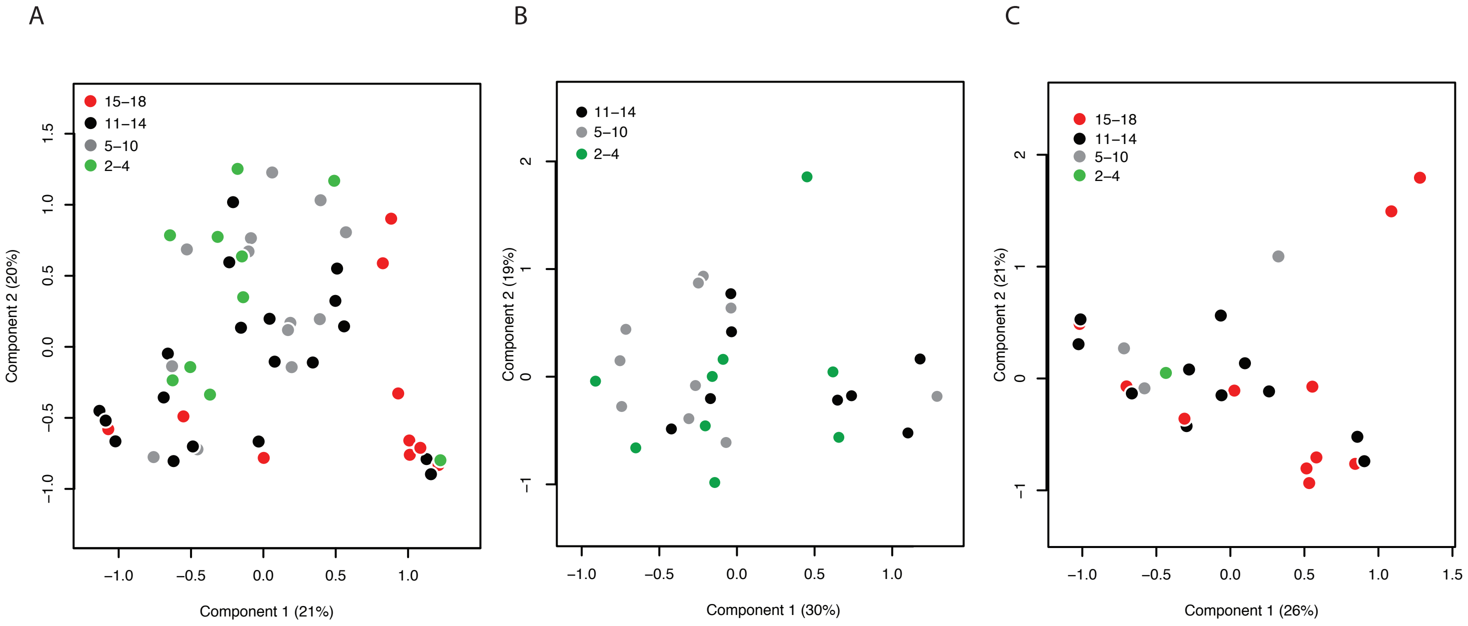

Supplementary figure 2. Effect of age on the microbial composition. **A)** When considering all subjects, age has a significant effect (p=0.006) on the microbial composition, contributing 11% to the microbial variation. However, when assessing the study groups **B)** non-IBD controls (p=0.11) and **C)** UC patients (p=0.76) separately, the age of the subject did not have a significant effect on the microbial composition.
